# Evaluating the Predictive Potential of an AI-Driven Deep Learning Model for Pneumonia-Associated Sepsis

**DOI:** 10.3390/jcm15062125

**Published:** 2026-03-11

**Authors:** Ki-Byung Lee, Chang Youl Lee, Jaewon Jang, Yeeun Jeong, Kyung Hyun Lee

**Affiliations:** 1 Department of Internal Medicine, Chuncheon Sacred Heart Hospital, Hallym University Medical Center, Chuncheon 24253, Republic of Korea; hallas79@hallym.or.kr; 2 AITRICS Corp., Seoul 06221, Republic of Korea; jjw2582@aitrics.com (J.J.); yeeun.jeong@aitrics.com (Y.J.); lkh256@aitrics.com (K.H.L.)

**Keywords:** sepsis, pneumonia, deep learning, clinical decision support system, early prediction

## Abstract

**Background**: Pneumonia-associated sepsis constitutes a significant portion of all sepsis cases and is a leading cause of sepsis-related morbidity and mortality. The clinical burden is especially pronounced in general ward settings, where delayed recognition can hinder timely intervention. This underscores the necessity for advanced tools that facilitate early detection. **Methods**: This retrospective, single-center study assessed an AI-driven deep learning model designed to predict in-hospital sepsis up to four hours in advance. We analyzed 7715 pneumonia cases identified through chest radiography or CT. The model’s performance was evaluated using AUROC, sensitivity, specificity, and lead time to sepsis onset and was compared against established scoring systems: NEWS, MEWS, SOFA, and qSOFA. Sepsis was defined according to the CDC Adult Sepsis Event criteria in alignment with Sepsis-3 guidelines. **Results**: The AI model exhibited strong performance in the early detection of sepsis among pneumonia patients, achieving an AUROC of 0.870, with a sensitivity of 76.7% and specificity of 84.1%. It significantly surpassed conventional scoring systems: NEWS (0.697), MEWS (0.661), SOFA (0.649), and qSOFA (0.678). Importantly, the model identified sepsis a median of 183 min earlier than recognition based on the operational definition. This lead-time advantage was consistent in the pneumonia cohort, where 18.3% of patients developed sepsis. **Conclusions**: The AI model demonstrated strong predictive capabilities for pneumonia-associated sepsis, facilitating earlier clinical decision-making. Integrating this model into EMR systems could be an effective strategy to enhance sepsis outcomes in general ward settings. Further prospective studies are needed to validate its effectiveness in real-time clinical applications.

## 1. Introduction

Sepsis is a life-threatening syndrome characterized by a dysregulated immune response to infection, leading to organ dysfunction and significant morbidity and mortality. Despite global advancements in diagnostic and therapeutic strategies, sepsis remains a global healthcare burden, affecting an estimated 49 million individuals annually [1].

Among the various etiologies, pneumonia-associated sepsis represents a substantial proportion and is notable for its respiratory complications and rapid progression to critical illness [2]. Early recognition and timely intervention are critical to prevent irreversible organ damage and improve outcomes [3,4]. When pneumonia progresses to sepsis, the presumed source of infection is typically pneumonia itself. This predictable trajectory enables early assessment of the adequacy of initial management, including the appropriateness of antibiotic escalation and timely ICU transfer. Such evaluations may facilitate earlier detection of sepsis progression and support more efficient allocation of critical care resources, such as ventilators and ICU beds.

The introduction of Sepsis-3 guidelines marked a significant milestone in the understanding and classification of sepsis, emphasizing early organ dysfunction detection through tools such as the Sequential Organ Failure Assessment (SOFA) [5]. However, this method was originally designed to assess the patient’s condition in ICU settings and is often limited by the need for laboratory tests and clinical evaluations, which may delay diagnosis, particularly in general ward settings. Similarly, conventional clinical scores like the Modified Early Warning Score (MEWS) and National Early Warning Score (NEWS) offer limited sensitivity and specificity for early sepsis detection, as they were not originally developed for sepsis-specific prediction but rather for general patient deterioration [6].

To address these limitations, the U.S. Centers for Disease Control and Prevention (CDC) introduced the Adult Sepsis Event (ASE) surveillance definition, which integrates suspected infection and concurrent organ dysfunction using routinely available electronic health record data [7]. This definition, originally developed by Rhee et al., has demonstrated strong concordance with the Sepsis-3 framework and has been widely adopted in large-scale observational studies [8,9].

Moreover, recent advancements in artificial intelligence (AI) have introduced a promising alternative for early sepsis prediction, especially in clinical settings where traditional scoring systems lack sufficient accuracy or timeliness. AI algorithms can process complex and heterogeneous clinical data—including vital signs, laboratory values, and electronic medical records—in real time to identify subtle, nonlinear patterns preceding clinical deterioration. This capability may enhance diagnostic accuracy and enable more timely clinical decision-making [10,11,12]. This study investigates the clinical efficacy of an AI-driven sepsis predictive model within 4 h, VitalCare-SEPsis (VC-SEPS), for the early detection of sepsis in patients with pneumonia, with the aim of supporting timely clinical decision-making and improving patient outcomes.

By utilizing retrospective real-world data combined with machine learning-based artificial intelligence technologies, this study aims to evaluate the feasibility of clinically meaningful early detection of pneumonia-associated sepsis. Such early recognition and timely intervention have the practical potential to significantly improve clinical outcomes, reduce morbidity and mortality, and alleviate healthcare burdens associated with pneumonia-associated sepsis [13].

Accordingly, this study evaluated the clinical utility of VC-SEPS, an AI-based deep learning model, for predicting sepsis within 4 h among hospitalized patients with pneumonia in general wards. We hypothesized that the VC-SEPS model, by integrating longitudinal Electronic Medical Record (EMR) data and capturing temporal dependencies, would demonstrate superior predictive performance compared to conventional scoring systems (NEWS, MEWS, SOFA, and qSOFA), thereby facilitating timely clinical intervention and informing broader clinical implementation.

## 2. Methods

### 2.1. Study Design and Setting

This retrospective cohort study included 7715 adult patients diagnosed with pneumonia based on chest X-ray or chest CT findings, selected from 43,147 general ward patients admitted to a teaching hospital in Chuncheon-si, Gangwon-do, Republic of Korea, between 1 January 2018 and 31 December 2022.

The predicted timing of sepsis using VC-SEPS algorithm (version 1.3.0; AITRICS, Seoul, Republic of Korea)was compared with the operational definition of sepsis from the U.S. CDC Toolkit for Adult Sepsis Surveillance, which defines sepsis based on suspected infection with concurrent organ dysfunction (ASE criteria). The performance of the AI model was also evaluated against established clinical deterioration prediction tools, including NEWS, MEWS, SOFA, and quick SOFA (qSOFA), for assessing sepsis-related symptoms. This study was conducted in accordance with the Declaration of Helsinki and approved by the Institutional Review Board (IRB) of the hospital (IRB No. 2023-03-007), with a waiver of informed consent due to its retrospective cohort design. The reporting of this study was informed by the principles of the TRIPOD (Transparent Reporting of a multivariable prediction model for Individual Prognosis or Diagnosis) statement to ensure a clear and transparent description of this model validation process.

### 2.2. Patient Selection and Data Collection

**Figure 1:**
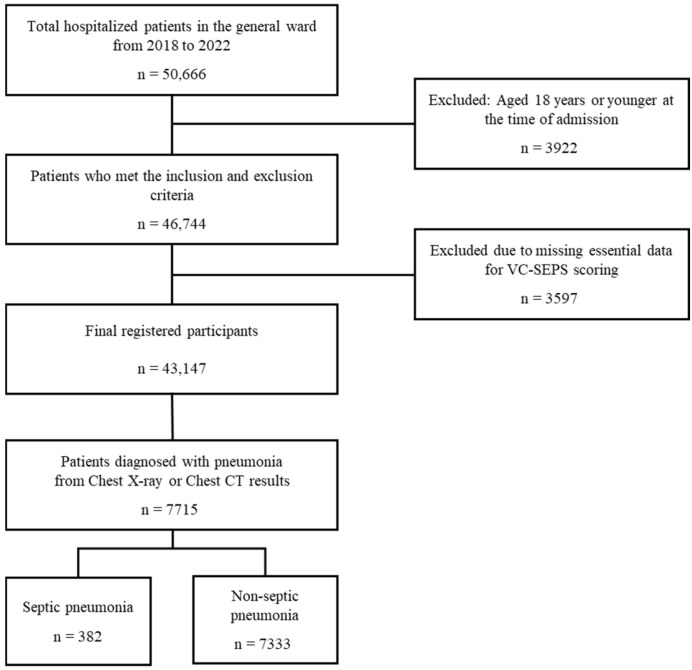
Flowchart of the patient selection process.

All patients aged 19 years and older who were admitted to the teaching hospital between 1 January 2018 and 31 December 2022 were initially included in the study. As shown in Figure 1, a total of 50,666 hospitalized patients in the general ward were screened from the teaching hospital’s Clinical Data Warehouse (CDW) during the 5-year data collection period. 46,744 patients met the inclusion and exclusion criteria and were registered for the study. Among the registered patients, 3597 were excluded due to missing essential data, which prevented the calculation of the VC-SEPS score, resulting in their removal from the analysis. Ultimately, 43,147 valid participants were included in the final dataset, and 7715 pneumonia patients were identified based on diagnostic findings from chest X-ray or chest CT imaging.

Patient data used to generate VC-SEPS scores were extracted from the Clinical Data Warehouse (CDW) and included vital signs (systolic and diastolic blood pressure, pulse rate, respiratory rate, body temperature, and oxygen saturation), laboratory results (total bilirubin, lactate, pH, sodium, potassium, creatinine, hematocrit, white cell count, bicarbonate, platelet count, and C-reactive protein), Glasgow Coma Scale (GCS) score, and age. Additional demographic and clinical data, such as blood culture results, diagnosis codes, medication records, chest X-ray and chest CT findings, as well as admission and discharge records, were also collected from the CDW to accurately identify and evaluate sepsis and pneumonia-associated sepsis.

### 2.3. Algorithm of Sepsis Prediction

The VC-SEPS is a deep learning-based prediction model integrated into the EHR system to analyze and process patient medical data in real time. This model is designed to detect sepsis in patients by issuing alerts up to 4 h prior to its clinical onset. It processes time-series data collected from routine clinical practice. Among the extracted features, vital signs such as systolic blood pressure (SBP), diastolic blood pressure (DBP), pulse rate (PR), respiratory rate (RR), and body temperature (BT) were utilized as core inputs for score calculation. Additionally, score generation incorporated the aforementioned selected panel of laboratory results, including pH, hematocrit, white blood cell count, platelet count, bilirubin, lactate, creatinine, sodium, potassium, bicarbonate, and C-reactive protein levels, along with oxygen saturation and GCS score. Missing values were imputed using the last observation carried forward (LOCF) method, and, as in general ward settings, where clinical measurements are obtained at irregular and relatively infrequent intervals, the last observed value represents the most clinically reasonable estimate of the patient’s current state. Normal values were used in cases where no prior data were available. The VC-SEPS model has been approved by the Korean Ministry of Food and Drug Safety (MFDS) for clinical use in general wards as an AI-based Clinical Decision Support System (CDSS). Its predictive performance and risk stratification capability for sepsis have been externally validated in hospitalized patients using real-world data in a prospective study [14].

The VC-SEPS is based on a bidirectional long short-term memory (biLSTM) neural network architecture, which was initially developed using intensive care unit (ICU) data to capture complex temporal dependencies in longitudinal EMR records [15]. The model is designed to integrate dynamic time-series features (e.g., vital signs and laboratory results) and static baseline features (e.g., demographics). To adapt this system for general ward deployment, several key modifications were implemented to address the data characteristics of non-ICU settings, such as lower sampling frequencies and irregular observation intervals [16]. In this general ward-optimized version, a robust last observation carried forward (LOCF) imputation strategy was employed to ensure continuous temporal input, which is essential for stable sequence processing within the biLSTM architecture. Furthermore, as detailed in our recent development study [16], the model’s weight optimization was refined to prioritize high-precision alerts in low-acuity environments. These technical enhancements allow VC-SEPS to provide stable and reproducible sepsis predictions by effectively processing bidirectional temporal information within sparse clinical data.

The 4 h prediction horizon refers to the predefined labeling framework used during model development, whereby prediction time points were labeled positive if sepsis recognition occurred within the subsequent 4 h. Lead time was defined as the time difference (minutes) between the operational recognition time and the first alert time point at which the model score exceeded the prespecified threshold, calculated per patient encounter.

### 2.4. Performance Evaluation Statistical Analyses

Demographic characteristics were compared between the sepsis and non-sepsis groups among both the total patient population and pneumonia patients. Categorical variables are presented as frequencies (percentages), while continuous variables are presented as mean ± standard deviation (SD) for normally distributed variables and median (interquartile range [IQR]) for non-normally distributed variables. Normality assumptions for continuous variables were assessed using the Shapiro-Wilk test.

The accuracy of the VC-SEPS model in predicting sepsis associated with pneumonia was assessed using the area under the receiver operating characteristic curve (AUROC). Additionally, the prediction speed of each model (NEWS, MEWS, SOFA, and qSOFA) was evaluated by analyzing their average prediction times. Statistical significance was determined at a *p*-value threshold of 0.05. All statistical computations were performed using Python version 3.11.5. The domain-adapted threshold (≥14) used for early detection analysis was determined in the validation cohort as the cut-off yielding the highest sensitivity among those achieving a specificity of at least 95% and was fixed prior to evaluation in the independent test dataset.

## 3. Results

### 3.1. Study Cases and Baseline Characteristics

The baseline demographic characteristics of the study participants are summarized in Table 1. The study population comprised 43,147 patients, of whom 2468 (5.72%) were classified as sepsis cases (Table 1a). For the pneumonia subpopulation, a total of 7715 patients were identified, with 1569 (20.33%) presenting with sepsis (Table 1b).

The overall mean age of the total patient cohort was 58.41 ± 18.42 years, with a significant difference between the sepsis group (71.59 ± 15.16 years) and the non-sepsis group (57.61 ± 18.29 years, *p* < 0.001; Table 1a). The gender distribution was comparable between males (52.41%) and females (47.59%). In the sepsis group, males were more prominently represented (54.46%) than females (45.54%, *p* = 0.036). Among pneumonia patients, the mean age was 67.02 ± 17.63 years, with the sepsis group being significantly older (72.81 ± 14.54 years) compared to the non-sepsis group (65.54 ± 18.03 years, *p* < 0.001; Table 1b). Gender distribution showed no statistically significant difference, with males accounting for 55.94% and females accounting for 44.06% overall (*p* = 0.354). The Charlson Comorbidity Index (CCI) was slightly higher in the sepsis group compared to the non-sepsis group. Additionally, overall patient mortality differed significantly between groups, with a higher mortality rate observed in the sepsis group compared with the non-sepsis group (Table 1a,b).

These findings, as summarized in Table 1, indicate that age may be a potential factor associated with sepsis in pneumonia patients, highlighting the importance of these characteristics in predictive modeling for sepsis risk.

### 3.2. Performance of VC-SEPS in Predicting Sepsis in Pneumonia Patients

**Figure 2:**
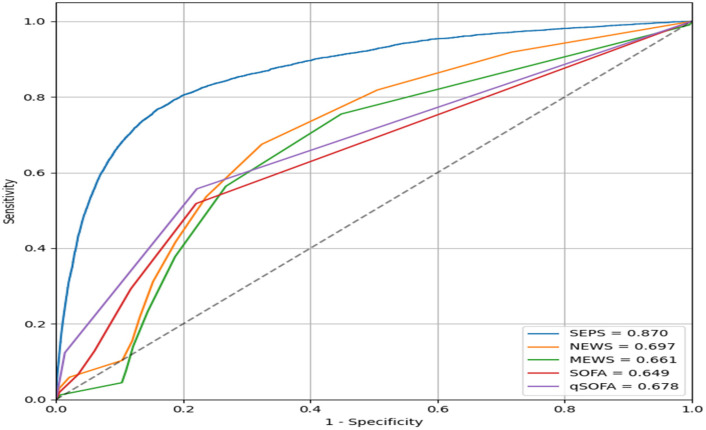
AUROC of VC-SEPS and traditional early warning scores for sepsis prediction in pneumonia patients. The dashed diagonal line represents the performance of a random classifier (AUC = 0.5), indicating no discriminative ability.

Based on data from 12,264 total admissions involving 7715 patients with pneumonia, VC-SEPS achieved an area under the receiver operating characteristic curve (AUROC) of 0.870, demonstrating superior discriminatory performance compared with conventional early warning scores. Specifically, the AUROC was 0.697 for NEWS, 0.661 for MEWS, 0.649 for SOFA, and 0.678 for qSOFA, all of which were significantly lower than that of VC-SEPS (*p* < 0.001) (Figure 2). These findings indicate that VC-SEPS more accurately identified pneumonia patients at risk of developing sepsis.

**Figure 3:**
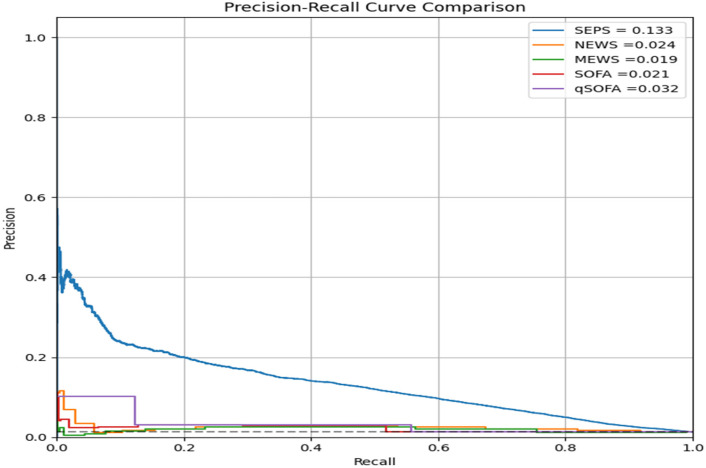
AUPRC of VC-SEPS and traditional early warning scores for sepsis prediction in pneumonia patients. The dashed horizontal line represents the baseline precision corresponding to the prevalence of the positive class.

In addition, precision–recall (PR) curve analysis was performed to further evaluate model performance in the context of class imbalance (Figure 3). The area under the PR curve (AUPRC) for VC-SEPS was 0.133, which was substantially higher than those of NEWS (0.024), MEWS (0.019), SOFA (0.021), and qSOFA (0.032). This indicates that VC-SEPS maintained higher precision across a wide range of recall values, reflecting improved identification of true-positive sepsis cases while limiting false-positive predictions among patients with pneumonia.

**Figure 4:**
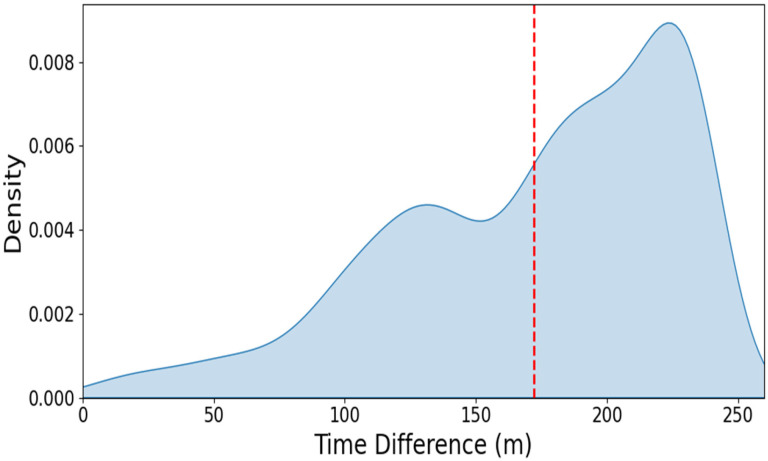
Time difference in predicting sepsis onset using VC-SEPS for pneumonia patients. The red dashed vertical line indicates the mean lead time of the first VC-SEPS alert relative to the clinical event.

Further analysis of early detection performance among patients with pneumonia showed that VC-SEPS achieved a sensitivity of 76.7% and a specificity of 84.1% at an optimal cut-off value of 11.87. Using a domain-adapted cut-off value of ≥14 for early detection, the mean lead time between VC-SEPS-predicted sepsis and sepsis recognition based on the operational definition was 183.0 min (95% CI, 181.8–184.2 min), corresponding to a median lead time of 183 min (Figure 4). Although the prediction horizon was set to 4 h, the observed lead time represents the empirical timing of threshold crossing within this window and may therefore be shorter than 240 min depending on patient trajectories and the selected operating threshold. Overall, the accuracy of early sepsis detection in pneumonia patients was 76.22%.

**Figure 5:**
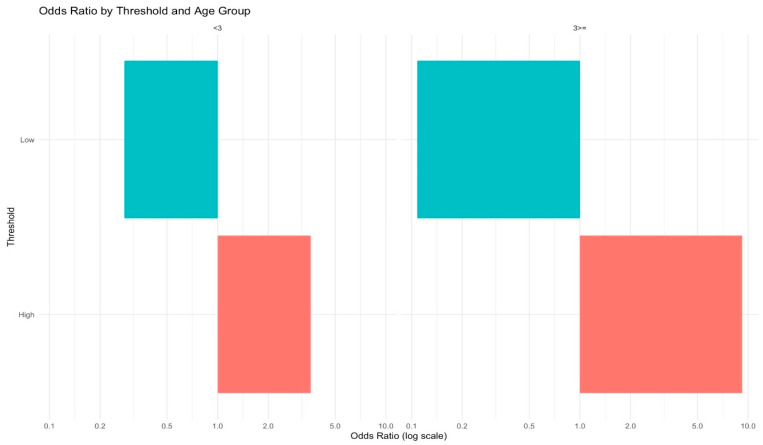
Odds ratios for septic pneumonia stratified by the Charlson Comorbidity Index (CCI). Patients with higher VC-SEPS scores had elevated odds in both groups, with greater amplification in those with CCI ≥ 3 (high-risk group). The cyan and coral bars represent the ‘Low’ (bottom 25th percentile) and ‘High’ (top 25th percentile) risk groups based on VC-SEPS scores, respectively. Odds ratios are presented on a log scale.

**Figure 6:**
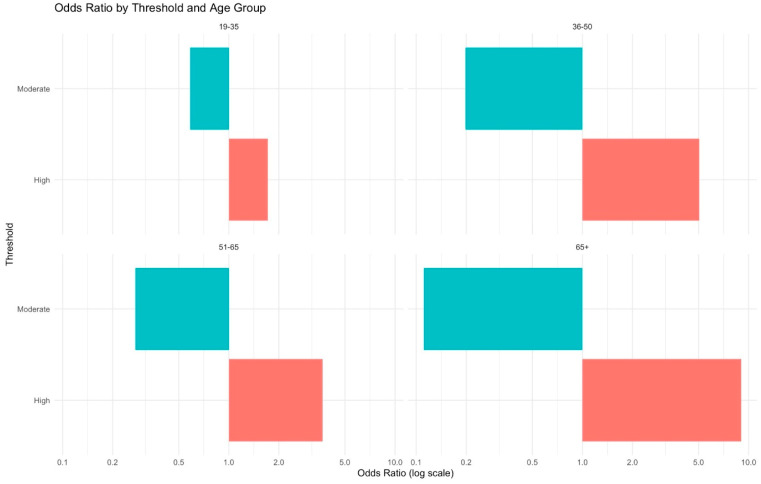
Odds ratios for septic pneumonia stratified by age. Across all age groups, patients with high VC-SEPS scores showed increased odds, with the risk progressively greater in older populations. Patients were categorized based on VC-SEPS score quartiles: Low (bottom 25th percentile, reference), Moderate (25th–75th percentile), and High (top 25th percentile). The cyan and coral bars represent the odds ratios for the Moderate and High groups, respectively. Odds ratios are presented on a log scale.

In addition to evaluating overall predictive performance, we examined patient-level risk modifiers. Higher VC-SEPS scores were consistently associated with increased odds of septic pneumonia across all strata. The risk amplification was more pronounced in patients with a Charlson Comorbidity Index (CCI) ≥3, which is commonly regarded as a high-risk threshold, and in older populations (Figure 5 and Figure 6). These findings suggest that comorbidity burden and advanced age may intensify the prognostic impact of the VC-SEPS score, highlighting its particular value in vulnerable patient groups.

## 4. Discussion

The results of this study highlight the potential of artificial intelligence (AI) in the early detection of sepsis, particularly among patients with pneumonia-associated sepsis [17,18]. In this study, VC-SEPS demonstrated superior discriminatory performance compared with conventional early warning scores in predicting sepsis among hospitalized patients with pneumonia in general ward settings. It should be noted that traditional clinical scoring systems, such as NEWS, MEWS, SOFA, and qSOFA, were primarily designed to assess a patient’s physiological severity at a specific cross-sectional point rather than for explicit temporal forecasting [9,19]. Consequently, comparing these static scores with a deep learning model optimized for early event prediction requires cautious interpretation. However, since these scores remain the current standard of care for monitoring patient deterioration in general wards, they serve as the most relevant benchmarks for the clinical utility of an AI-based early warning system [20]. While traditional scores such as NEWS, MEWS, SOFA, and qSOFA were developed primarily to detect general clinical deterioration or established organ dysfunction, VC-SEPS integrates high-dimensional temporal patterns from routinely collected clinical data, enabling earlier identification of subtle physiological changes preceding overt sepsis.

The primary aim of this study was to evaluate the added clinical value of VC-SEPS in comparison to established scoring systems used in routine practice. Beyond these conventional tools, it is imperative to position VC-SEPS within the current paradigm of AI-driven sepsis prediction. Recent advancements have shifted toward complex architectures, such as transformer-based models that utilize self-attention mechanisms to capture intricate temporal dependencies [21,22]. Furthermore, the clinical field has reached a milestone with the FDA clearance of platforms like the Sepsis ImmunoScore (Prenosis, Chicago, IL, USA), which incorporates clinical data with biological markers to enhance diagnostic precision [23]. While these state-of-the-art (SOTA) approaches show promise, their utility in general ward settings is frequently constrained by the requirement for high-resolution temporal data or specialized laboratory parameters. In contrast, the biLSTM-based architecture of VC-SEPS is optimized to maintain predictive robustness by utilizing a minimal set of essential variables from intermittent and irregularly sampled EMR data. Furthermore, we acknowledge that the present study does not include a direct comparison with other contemporary neural network-based sepsis prediction models. However, it is worth noting that our focus was primarily on evaluating the clinical performance of an existing regulatory-authorized AI software (VC-SEPS) in the pneumonia population, rather than benchmarking multiple competing architectures. The biLSTM-based architecture underlying VC-SEPS was developed when LSTM-based approaches were the predominant paradigm for clinical time-series modeling. Nevertheless, we view the current validated architecture as a robust baseline for clinical deployment, and future iterations of our system may incorporate attention-based mechanisms to further enhance predictive performance as data resolution improves. Ultimately, our findings suggest that VC-SEPS provides a feasible and scalable solution that yields performance comparable to current SOTA benchmarks while ensuring clinical applicability in resource-limited ward environments.

Notably, the observed performance advantage of VC-SEPS was achieved in a clinically challenging population—patients with pneumonia admitted to general wards—where early signs of sepsis are often nonspecific and may overlap with baseline respiratory abnormalities. In this context, the ability of VC-SEPS to provide early and accurate identification of patients at risk of sepsis suggests meaningful potential for supporting timely escalation of care, including antibiotic optimization and ICU triage, prior to irreversible organ dysfunction.

These results suggest that the model is effective in identifying a significant proportion of true sepsis cases while minimizing false positives, a key consideration for clinical implementation. Importantly, this balance between sensitivity and specificity is particularly relevant in general ward environments, where excessive false alarms may contribute to alert fatigue and limit the clinical usefulness of early warning systems. The VC-SEPS model demonstrated strong predictive performance, with an AUROC of 0.870, and reduced the time to sepsis prediction by a median of 183 min compared with conventional diagnostic methods based on operational surveillance definitions. This lead-time advantage corresponds to a clinically meaningful window during which early reassessment and intervention may be initiated before overt organ dysfunction develops. These findings add to the growing evidence that machine learning algorithms can provide timely and actionable clinical insights, potentially improving decision-making and outcomes in acute and critical care settings [24].

Although VC-SEPS demonstrated strong performance in this single-center cohort, external validation across diverse institutions and healthcare environments remains essential to confirm generalizability and robustness. Prospective multicenter studies are needed to determine whether earlier identification of high-risk patients translates into tangible improvements in clinically meaningful outcomes, including reduced organ dysfunction, shorter hospital stays, and lower mortality.

In real-world practice, AI-based predictive models may be integrated into electronic health record systems to provide real-time risk alerts that augment, rather than replace, clinical judgment. The potential clinical benefit of such systems lies in facilitating earlier reassessment, timely initiation of sepsis bundles, and appropriate escalation of care. However, successful implementation requires careful threshold selection, seamless workflow integration, and proactive mitigation of alert fatigue to maintain clinician trust and usability.

Furthermore, disparities in digital infrastructure, data availability, and institutional resources may limit the deployment of predictive systems in resource-limited settings. These considerations underscore the importance of flexible, context-adapted implementation strategies and continuous performance monitoring following deployment.

Regarding the recent emergence of large multimodal models (LMMs), it is crucial to distinguish the strategic orientation of the VC-SEPS system. While LMMs process vast, heterogeneous datasets, their practical deployment is often constrained by high computational demands. Sepsis occurs universally, including in diverse clinical settings where only universally measured and easily retrievable variables from Electronic Medical Records (EMRs) are available. Our approach prioritizes clinical universality and resource-agnostic versatility by focusing on a lightweight, highly efficient architecture that delivers robust performance using standardized features routinely collected in everyday practice. This ensures that the timely identification of high-risk patients remains possible even in hospitals with limited technological density. By centering on functional scalability rather than technological complexity, VC-SEPS effectively addresses unmet medical needs in global healthcare, providing a scalable and context-adapted solution to improve outcomes in both tertiary centers and resource-limited environments. While the emergence of large multimodal models (LMMs) offers a promising future for integrating diverse clinical data, their practical deployment remains hindered by high computational demands and regulatory complexities. In contrast, VC-SEPS provides an immediate, deployable solution by focusing on a lightweight, efficient architecture optimized for standardized EHR data. Thus, our approach and LMMs can be viewed as complementary: the former addresses current clinical scalability and regulatory clarity, while the latter represents a long-term direction for multidimensional clinical AI research.

Although the absolute area under the precision–recall curve (AUPRC) values appeared numerically modest, this finding should be interpreted in the context of the inherent class imbalance characteristic of sepsis prediction in general ward populations. Sepsis remains a relatively infrequent outcome among hospitalized patients with pneumonia, resulting in low baseline precision across all predictive models. In such settings, absolute AUPRC values are expected to be low, and relative improvements between models are more clinically informative. Importantly, VC-SEPS demonstrated a substantially higher AUPRC compared with all conventional early warning scores, indicating improved precision across a wide range of recall levels and a reduced burden of false-positive alerts, which is critical for real-world clinical implementation.

A major challenge in sepsis management is the difficulty of controlling the presumed source of infection, which complicates prediction and timely intervention. In pneumonia-to-sepsis progression, however, the source is predefined, providing an opportunity to anticipate deterioration through optimized pneumonia management. This may enable earlier antibiotic escalation, preemptive ICU transfer, and more efficient allocation of critical care resources. The VC-SEPS model showed high sensitivity and specificity, demonstrating performance advantages over conventional early warning scores such as MEWS and NEWS [6], underscoring its clinical utility in supporting earlier and more targeted interventions.

Although a statistically optimal cut-off value was identified through receiver operating characteristic analysis, a higher, domain-adapted threshold (≥14) was applied for early detection analyses to prioritize clinical specificity and mitigate alert fatigue. In general ward environments, excessive false-positive alerts may undermine clinician trust and reduce the effectiveness of clinical decision support systems. The adoption of a conservative cut-off reflects a deliberate balance between sensitivity and specificity, aiming to generate alerts that are more likely to prompt meaningful clinical action rather than indiscriminate notifications.

Importantly, the model was trained using data labeled according to operational criteria from the CDC Sepsis Toolkit and benchmarked against the Sepsis-3 framework, including SOFA scoring [5,25]. This dual-framework approach strengthens the reliability of the model and enhances its adaptability to evolving clinical standards. By using real-world retrospective data, this study aimed to reflect the performance of AI under practical clinical conditions rather than hypothetical scenarios.

This study has several limitations. First, SEPS thresholds were defined using quartiles, which are suitable for exploratory analyses but lack direct clinical validation. Second, although stratified analyses indicated stronger associations in high-CCI and older patients, we did not formally test for statistical interaction, and residual confounding by baseline risk cannot be excluded. Finally, the retrospective design and single-cohort setting limit the generalizability of the findings. Prospective, multi-center validation will be essential to confirm real-time performance and assess integration into routine workflows.

In conclusion, VC-SEPS represents a meaningful advance in the early detection of pneumonia-associated sepsis, demonstrating superior predictive performance compared with conventional methods and providing clinically actionable insights within critical timeframes. By aligning with both the CDC Sepsis Toolkit and Sepsis-3 guidelines [5,25], the model exemplifies the integration of advanced AI with established clinical frameworks. Further interdisciplinary research, including the incorporation of biomarkers, imaging, and real-time physiological data [26,27], will be required to refine predictive algorithms and expand their clinical utility.

## 5. Conclusions

In patients with pneumonia-associated sepsis, the VC-SEPS model predicted sepsis onset on average 3 h earlier than diagnoses based on operational sepsis definitions. The model also demonstrated superior performance compared to conventional clinical scores, such as NEWS and MEWS, in predictive accuracy. Further prospective studies are warranted to investigate factors contributing to the progression of pneumonia-associated sepsis and to expand relevant predictive categories, ultimately enabling earlier interventions and improving clinical outcomes.

## Figures and Tables

**Table 1:** Baseline characteristics of the study population.

(a) Total Patient Population									
		Subject-Wise				Record-Wise			
		Overall	Non-Sepsis	Sepsis	*p*-Value	Overall	Non-Sepsis	Sepsis	*p*-Value
N		43,147	40,679	2468		72,322	66,448	5874	
Age (years)		58.41 (18.42)	57.61 (18.29)	71.59 (15.16)	<0.001	60.50 (17.56)	59.87 (17.50)	69.70 (15.51)	<0.001
Gender	M	22,615 (52.41%)	21,271 (52.29%)	1344 (54.46%)	0.036	37,656 (52.07%)	34,508 (51.93%)	3148 (53.59%)	0.019
	F	20,532 (47.59%)	19,408 (47.71%)	1124 (45.54%)		34,666 (47.93%)	31,940 (48.07%)	2726 (46.41%)	
CCI		1.96 (1.78)	1.86 (1.72)	3.49 (1.86)	<0.01	2.27 (1.85)	2.17 (1.81)	3.40 (1.91)	<0.001
Mortality		1525 (3.6%)	773 (1.91%)	752 (31.44%)	<0.001	1525 (2.1%)	773 (1.17%)	752 (12.97%)	<0.001
**(b) Pneumonia Patient Population**									
		**Subject-Wise**				**Record-Wise**			
		**Overall**	**Non-Sepsis**	**Sepsis**	***p*** **-Value**	**Overall**	**Non-Sepsis**	**Sepsis**	***p*** **-Value**
**N**		**7715**	**6146**	**1569**		**12,264**	**9520**	**2744**	
Age (years)		67.02 (17.63)	65.54 (18.03)	72.81 (14.54)	<0.001	68.03 (16.67)	66.76 (16.98)	72.44 (14.76)	<0.001
Gender	M	4316 (55.94%)	3422 (55.68%)	894 (56.98%)	0.354	6777 (55.26)	5235 (54.99)	1542 (56.20%)	0.263
	F	3399 (44.06%)	2724 (44.32%)	675 (43.02%)		5487 (44.74)	4285 (45.01)	1202 (43.80%)	
CCI		2.91 (1.93)	2.72 (1.91)	3.65 (1.83)	<0.001	3.10 (1.93)	2.92 (1.91)	3.69 (1.86)	<0.001
Mortality		1251 (16.22%)	589 (9.58%)	662 (42.19%)	<0.001	1251 (10.20%)	589 (6.19%)	662 (24.13%)	<0.001

## Data Availability

The data presented in this study are available upon request from the corresponding author. The data are not publicly available due to privacy and ethical restrictions related to patient medical records.
